# Computational fluid dynamics analysis on endoscopy of main left coronary artery: An application of applied mathematics

**DOI:** 10.1016/j.heliyon.2024.e26628

**Published:** 2024-02-21

**Authors:** Salman Akhtar, Zahir Hussain, Hassan Ali Ghazwani, Showkat Ahmad Lone, Emad A. Az-Zo'bi

**Affiliations:** a Department of Mathematics, Quaid-i-Azam University 45320, Islamabad, 44000, Pakistan; b School of Engineering, University of Leicester, Leicester, LE1 7RH, UK; c Department of Mechanical Engineering, Faculty of Engineering, Jazan University, P.O. Box 45124, Jazan, Kingdom of Saudi Arabia; d Department of Basic Sciences, College of Science and Theoretical Studies, Saudi Electronic University, Riyadh, 11673, Kingdom of Saudi Arabia; e Departmennt of Mathematics, Mutah University, Al Karak, Jordan

**Keywords:** Endoscopy, Coronary artery blood flow, Stenosis, Non-Newtonian model

## Abstract

The endoscopy of a coronary arterial segment having a symmetric emergence of plaque at its innermost region is numerically modeled via computational fluid dynamics toolbox Open-FOAM. The considered left coronary artery for this model has a radius of 2 mm and span of 10 mm. The formation of plaque inside the artery that is a stenosis has length 2 mm and height 0.82 mm. The catheter used for this analysis has a diameter of 1 mm with a balloon over it with a height of 0.53 mm. The blood flow rate considered for this analysis has a range 2.00 ml/s to 2.50 ml/s. The fluid under consideration for this endoscopy review is the non-Newtonian Casson model. The mesh illustrations are arranged for the proposed model with numerical simulations of velocity, pressure profile and streamlines. The narrow channel formed due to assembly of stenosis and balloon over catheter inside this arterial segment has developed some swirling flow profile with turbulence effects just after the flow leaves the stenosis plus balloon region. Although this disturbance caused due to narrowing of channel has made the flow slightly turbulent, the flow eventually leaves the arterial segment again as a laminar flow. To cure coronary artery disease, catheterization, and balloon dilation of stenosed arteries is performed to locate the position and shape of stenosis. A catheter is inserted inside the body through a minor cut and then it is moved inside arteries to place it exactly at the stenosis location. A balloon is placed at front of that catheter and the stenosed region can be opened wide by using balloon dilation.

## Nomenclature

${ẟ}^{*}$: Height of stenosis
n: Shape formation of stenosis factor
ζ: Viscosity of Casson fluid
${\bar{\dot{\gamma }}}_{ij}$: Shear strain rate
m: Index of consistency
${\bar{S}}_{ij}$: Yield stress
$\bar{t}$: time
$\bar{p}$: pressure
cR: Radius of catheter
$\left(\bar{r},\bar{z}\right)$: Cylindrical Coordinates
$\left(\bar{u},\bar{w}\right)$: Velocity field
a: Location of stenosis (starting point)
L: Length of arterial segment
R: Arterial radius of normal section
b: Stenosis length
ρ: Fluid density
$\bar{z}$: Length of axial coordinate
CFD: Computational fluid dynamics

## 1 Introduction

Coronary artery disease is a common heart disease that is the major cause of death around the world. Some plaque is assembled inside the coronary artery due to smoking, fats and oils or maybe sometimes due to lack of exercise and not having any other physical activity. This results in a lower supply of oxygen, nutrients, blood cells etc to the heart muscles that causes pain in chest, slower breathing or in severe cases a heart attack is the result of this disease. Many researchers have added fine literature work in this regard and provided an innovative blood flow analysis inside such stenosed arteries with endoscopy. Halder [1] had provided the blood flow interpretations through various stenosis segments. He had considered various formations of stenosis and their resistance effects. Chakravarty et al. [2] had numerically illustrated hemodynamics through an arterial control volume featuring a mild plaque formation. They had computed the numerical solutions via finite difference scheme. Ku [3] had modeled the unsteady flow phenomenon of blood flow through an arterial segment having a stenosis and shown that how turbulence occurs due to stenosis formation and results in a flow reduction. Mandal [4] had illustrated and examined the hemodynamics through a symmetric stenosis arterial section of converging plus diverging narrow artery. He had considered the unsteady blood flow phenomenon by taking generalized power law (non-Newtonian) representation and computed numerical simulation work via FDM. Ponalagusamy [5] had taken various features of plaque assembly in his analysis of hemodynamics through an arterial control volume. Ellahi et al. [6] had considered the non-Newtonian micro-polar fluid model for an arterial control volume featuring stenosis. They had provided exact (analytical) mathematical results in their analysis with graphical interpretations. Some of the latest research articles on hemodynamics rheology via a fine artery control volume featuring various stenosis shapes are referred to as [[7], [8], [9], [10], [11]].

The endoscopy analysis on hemodynamics via an arterial control volume featuring various stenosis is also covered by many recent researchers. Abdelsalam and Bhatti [12] had exemplified the endoscopy analysis of hemodynamics mechanism by availing the non-Newtonian Prandtl model and provided the Homotopy solutions for the problem. Akbar and Nadeem [13] had computed exact analytical solutions on endoscopy analysis of a bio-viscous non-Newtonian fluid. Akram and Akbar [14] had mathematically exemplified the non-Newtonian (Carreau fluid) representation for an endoscopy analysis inside an inclined conduit. Mekheimer et al. [15] had conveyed the endoscopy interpretation on blood flow through an overlapping arterial stenosis section. They had considered the blood flow analysis for a non-Newtonian Jeffrey fluid. Karmakar et al. [16] illustrated and examined the endoscopic operations on hemodynamics via an arterial cross-section under electro-osmotic impact. He calculated and interpreted analytical (exact) mathematical outcomes for the analysis. Some latest research work that considers the endoscopic operations on blood flow, nanofluid flow, electroosmotic pumping, overlapping stenosis is given as [[17], [18], [19], [20], [21], [22], [23], [24], [25], [26], [27], [28], [29]].

We have carefully reviewed the existing literature and provide here a CFD model on endoscopy of blood flow rheology inside an arterial segment featuring uniform stenosis. The non-Newtonian rheology of hemodynamics is tackled by availing the Casson representation for this research work. A catheter is inserted inside this arterial segment that catheter also has a balloon attached on its tip. Then the balloon over this endoscope is positioned at the underlying spot of plaque to compute the numerical simulations. The numerical simulations work on the endoscopy analysis of this stenosed arterial section is provided for the velocity and pressure profile. Streamlines are included that depict some interesting swirling and turbulent flow outcomes. Although the overall flow profile remains laminar at both entering and leaving regions of arterial segment just a slight turbulence is noted as the flow leaves the stenosis section.

## 2 Mathematical model

**Fig. 1:**
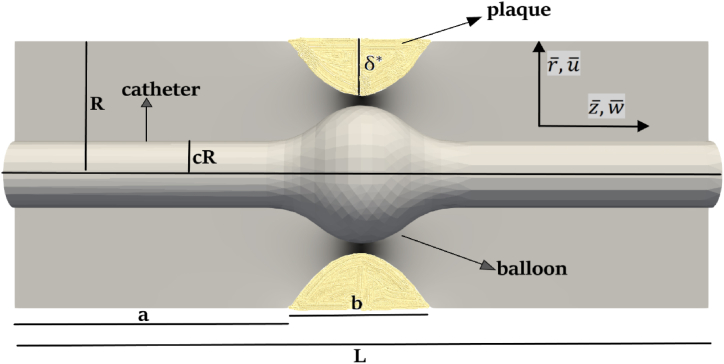
Geometrical model of endoscopy of a stenosed coronary arterial segment.

The mathematical model is provided for the endoscopy of a stenosed arterial segment of coronary artery. The catheter placed inside the artery for endoscopic purposes has a balloon placed on its tip. The unsteady, laminar, incompressible, and non-Newtonian flow is assumed for the blood flow problem. The geometrical diagram of the presented model is given in figure (1).

The considered segment of coronary artery has a well-formed symmetric stenosis at the innermost spot that is contemplated by the underlying equations referred as [6,14].

(1) $$\bar{\eta }\left(\bar{z}\right)=\left\{ \begin{array}{c} R\left[1-\kappa \left\{b^{n-1}\left(\bar{z}-a\right)-{\left(\bar{z}-a\right)}^{n}\right\}\right],a\leq \bar{z}\leq a+b\\ R\;otherwise \end{array}\right.\text{,}$$

The endoscopy of this coronary arterial segment is considered by inserting a catheter that has a balloon over it inside the artery. The catheter that has a balloon on its tip is exactly placed at the location of plaque assembly so that the plaque may be removed by the expansion of balloon over the catheter. The mathematical expression that contemplates the catheter plus balloon structure is referred as follows [6,14].

(2) $$\bar{∊}\left(\bar{z}\right)=\left\{ \begin{array}{c} R\left[c+f_{1}\left(\bar{z}\right)\right],a\leq \bar{z}\leq a+b\\ cR\;otherwise \end{array}\right.\text{,}$$

The balloon structure and shape depend on the function $f_{1}\left(\bar{z}\right)$ whereas κ=ẟ*Rbnnnn−1n−1..

A non-Newtonian flow model is reviewed for this endoscopy of arterial blood flow problem. The governing equations are referred as [[18], [19], [20]].

(3) $$\frac{\partial \bar{u}}{\partial \bar{r}}+\frac{\bar{u}}{\bar{r}}+\frac{\partial \bar{w}}{\partial \bar{z}}=0\text{,}$$

(4) $$\rho \left(\frac{\partial \bar{u}}{\partial \bar{t}}+\bar{u}\frac{\partial \bar{u}}{\partial \bar{r}}+\bar{w}\frac{\partial \bar{u}}{\partial \bar{z}}\right)=-\frac{\partial \bar{p}}{\partial \bar{r}}+\frac{1}{\bar{r}}\frac{\partial }{\partial \bar{r}}\left(\bar{r}{\bar{S}}_{rr}\right)+\frac{\partial }{\partial \bar{z}}\left({\bar{S}}_{rz}\right)\text{,}$$

(5) $$\rho \left(\frac{\partial \bar{w}}{\partial \bar{t}}+\bar{u}\frac{\partial \bar{w}}{\partial \bar{r}}+\bar{w}\frac{\partial \bar{w}}{\partial \bar{z}}\right)=-\frac{\partial \bar{p}}{\partial \bar{z}}+\frac{1}{\bar{r}}\frac{\partial }{\partial \bar{r}}\left(\bar{r}{\bar{S}}_{rz}\right)+\frac{\partial }{\partial \bar{z}}\left({\bar{S}}_{zz}\right)\text{,}$$

The admissible set of boundary conditions for this physical model is as follows.

(6) $$\begin{array}{c} \bar{w}=0\;at\;\bar{r}=\bar{∊}\left(\bar{z}\right)\text{,}\\ \bar{w}=0\;at\;\bar{r}=\bar{\eta }\left(\bar{z}\right)\text{,} \end{array}$$

These are no slip boundary conditions which show that the fluid particles in contact with wall and catheter have zero velocity. The appropriate non-Newtonian model that is availed for this work is the Casson fluid model and the basic expression to consider the extra stress tensor is referred as [19,20].

(7) $$\sqrt{\zeta }=\sqrt{\frac{{\bar{S}}_{ij}}{{\bar{\dot{\gamma }}}_{ij}}}+\sqrt{m}\text{,}$$

The explicit form of extra stress tensors is given as [19,20].

$${\bar{S}}_{rr}=\frac{\partial \bar{u}}{\partial \bar{r}}\left(\zeta +m-2\sqrt{\zeta m}\right),{\;\bar{S}}_{zz}=\frac{\partial \bar{w}}{\partial \bar{z}}\left(\zeta +m-2\sqrt{\zeta m}\right)\text{,}$$

$${\;\bar{S}}_{rz}=\frac{1}{2}\left(\frac{\partial \bar{u}}{\partial \bar{z}}+\frac{\partial \bar{w}}{\partial \bar{r}}\right)\left(\zeta +m-2\sqrt{\zeta m}\right)\text{,}$$

## 3 Numerical solution

**Fig. 2:**
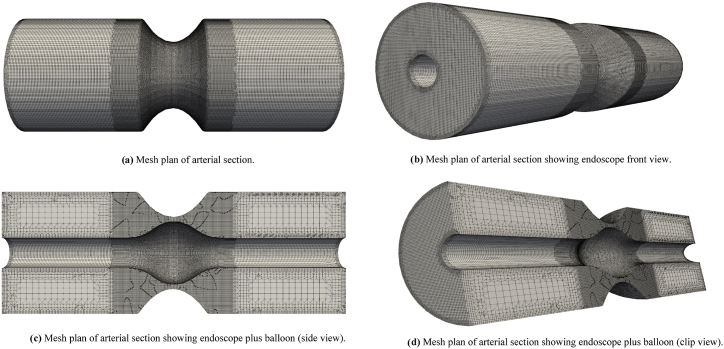
Fig. (2-a) Mesh plan of arterial section. Fig. (2-b) Mesh plan of arterial section showing endoscope front view. Fig. (2-c) Mesh plan of arterial section showing endoscope plus balloon (side view). Fig. (2-d) Mesh plan of arterial section showing endoscope plus balloon (clip view).

The mesh diagrams are provided in figures (2a-2d). It is evident that the cross-section of arterial segment containing the plaque and balloon is refined more carefully to get some good simulation results. The corners of the considered artery plus the corners of endoscope are also refined more carefully, that is evident from mesh diagrams. The simulation results and the mesh diagrams are obtained by utilizing the freely available C++ toolbox Open FOAM. The backend working of this program is based on the numerical method so called finite volume method that discretizes the whole considered volume into tiny units and solves the pertinent partial differential equation for each tiny volume part and computes the solution. The iterations are run from t=1 to t=10 for the case. The value of Δt is set to 0.001 and the maximum value of courant number is set to remain below or equal to 0.5. The algorithm pimple is used to handle the problem under consideration with some modifications to take laminar non-Newtonian flow of Casson fluid.

## 4 Results and discussion

**Fig. 3:**
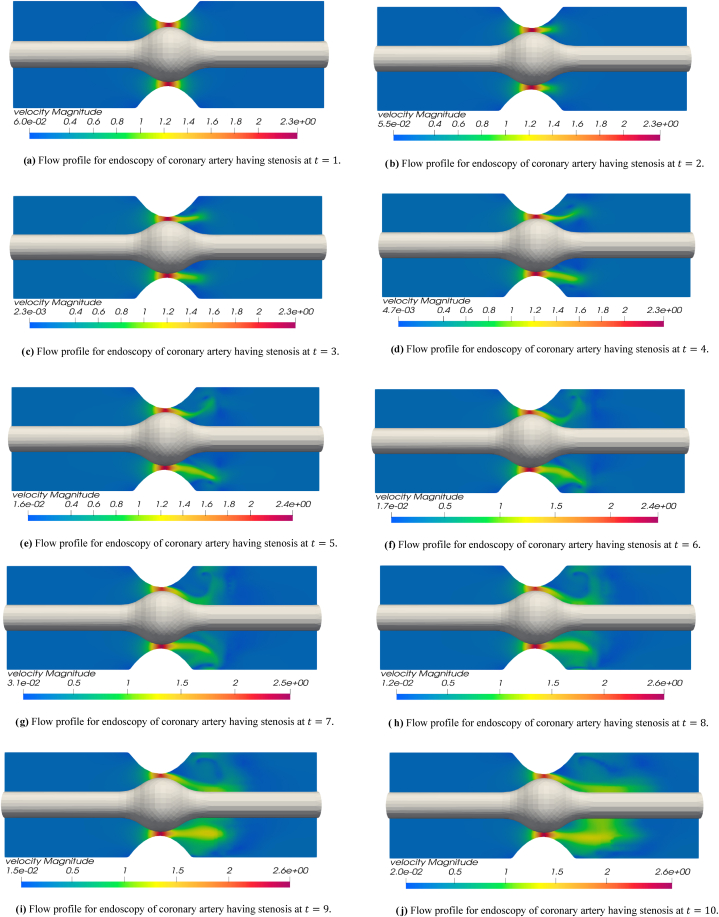
Fig. (3a) Flow profile for endoscopy of coronary artery having stenosis at t=1.,Fig. (3b) Flow profile for endoscopy of coronary artery having stenosis at t=2., Fig. (3c) Flow profile for endoscopy of coronary artery having stenosis at t=3., Fig. (3d) Flow profile for endoscopy of coronary artery having stenosis at t=4., Fig. (3e) Flow profile for endoscopy of coronary artery having stenosis at t=5., Fig. (3f) Flow profile for endoscopy of coronary artery having stenosis at t=6., Fig. (3g) Flow profile for endoscopy of coronary artery having stenosis at t=7., Fig. (3h) Flow profile for endoscopy of coronary artery having stenosis at t=8., Fig. (3i) Flow profile for endoscopy of coronary artery having stenosis at t=9., Fig. (3j) Flow profile for endoscopy of coronary artery having stenosis at t=10..

**Fig. 4:**
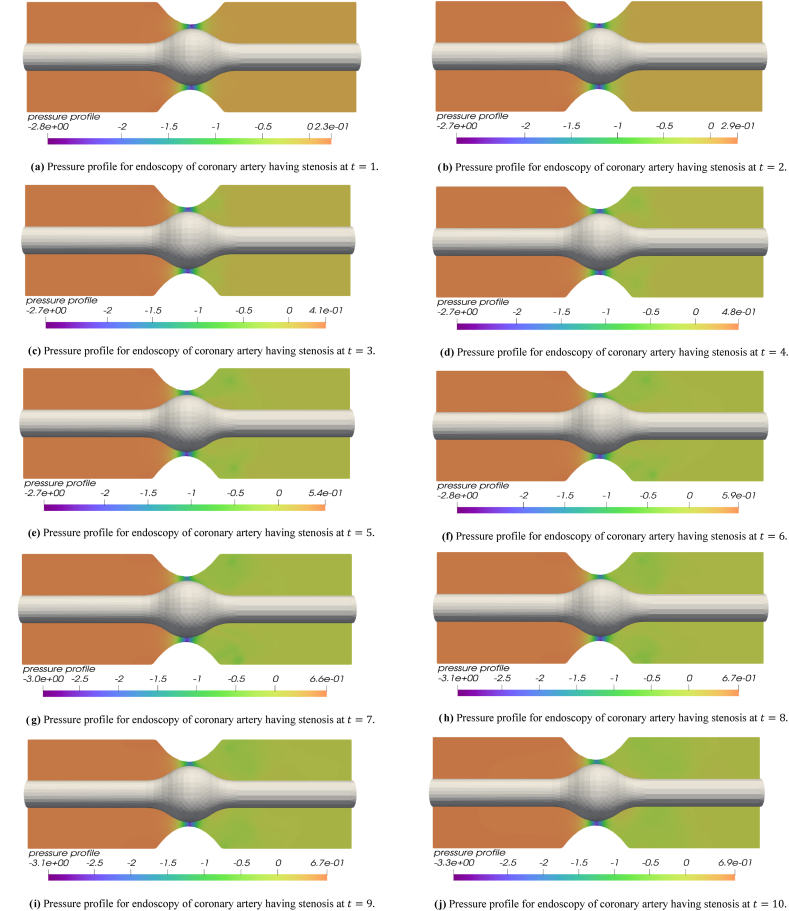
Fig. (4a) Pressure profile for endoscopy of coronary artery having stenosis at t=1., Fig. (4b) Pressure profile for endoscopy of coronary artery having stenosis at t=2., Fig. (4c) Pressure profile for endoscopy of coronary artery having stenosis at t=3., Fig. (4d) Pressure profile for endoscopy of coronary artery having stenosis at t=4., Fig. (4e) Pressure profile for endoscopy of coronary artery having stenosis at t=5., Fig. (4f) Pressure profile for endoscopy of coronary artery having stenosis at t=6., Fig. (4g) Pressure profile for endoscopy of coronary artery having stenosis at t=7., Fig. (4h) Pressure profile for endoscopy of coronary artery having stenosis at t=8., Fig. (4i) Pressure profile for endoscopy of coronary artery having stenosis at t=9., Fig. (4j) Pressure profile for endoscopy of coronary artery having stenosis at t=10..

**Fig. 5:**
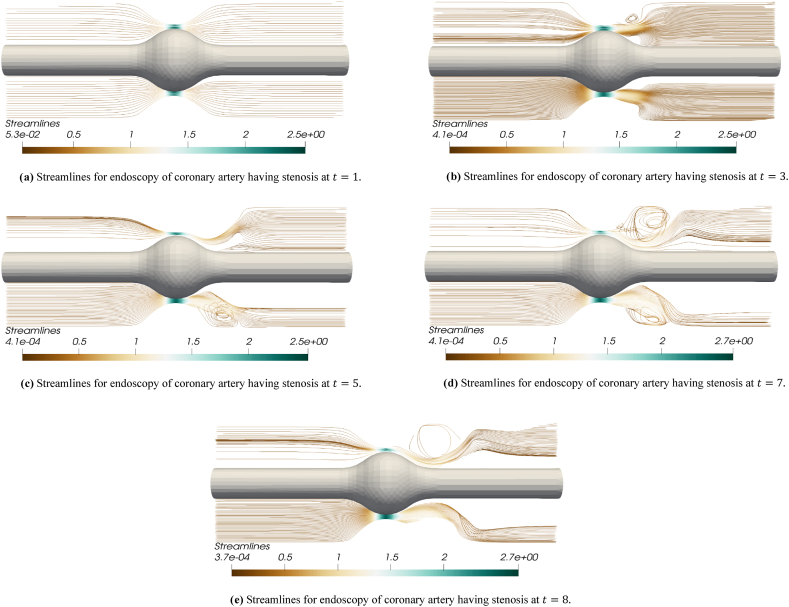
Fig. (5a) Streamlines for endoscopy of coronary artery having stenosis at t=1., Fig. (5b) Streamlines for endoscopy of coronary artery having stenosis at t=3., Fig. (5c) Streamlines for endoscopy of coronary artery having stenosis at t=5., Fig. (5d) Streamlines for endoscopy of coronary artery having stenosis at t=7., Fig. (5e) Streamlines for endoscopy of coronary artery having stenosis at t=8..

The blood flow simulations are exemplified for the iterations t=1 to t=10. Figures (3a-3j) disclose the numerical outcomes for the simulations of hemodynamics profile for the endoscopy of a stenosed section of coronary artery. In Fig. (3a) for t=1, the blood flow profile is almost smooth throughout the arterial segment however a high flow profile is observed at the tip point of stenosis. The flow achieves high velocity values as it enters the narrow region containing balloon and stenosis while velocity values decline as the flow leaves this region. In Fig. (3b) for t=2, one can see the developing flow profile for the blood leaving the balloon and stenosis section. In Figs. (3c, 3d) for t=3 and t=4, the blood flow profile leaving the stenosis plus balloon segment is more evident and a clear stream can be observed leaving the stenosis section. In remaining Figs. (3e-3j) for t=5 to t=10, it is observed that a slightly swirling flow is starting to develop just after the blood leaves the stenosis and balloon region. In Figs. (3h, 3i) for t=8 and t=9, a complete circular rotation of flow or swirling flow can be observed in upper section of artery just where the blood crosses the stenosis plus balloon region. In Fig. (3j) at t=10, the flow can be seen leaving the stenosis and balloon section at a high velocity. In all the velocity graphs for t=1 to t=10, initially the velocity is same throughout the arterial segment but only a higher flux is observed at the tip of balloon and plaque assembly while the fluid can be seen leaving the balloon plus stenosis section at a higher velocity as the iterations move towards t=10. The reason to observe the swirling flow profile just after the blood leaves the stenosis section is due to the occurrence of turbulence that happens naturally in the region where stenosis lies in real life problems. Here in this problem not only stenosis but also a balloon lies in the same region that makes the channel narrow enough to cause a slight turbulence effect and swirls in the flow. The turbulence and swirling effect happening due to stenosis and balloon made narrow region is also evident in streamline graphs that will be explained later. In Figures (4a-4j), the pressure profile is depicted for the endoscopy analysis of a stenosed artery blood flow problem. These figures provide the respective iterations for t=1 to t=10. In Fig. (4a) at t=1, a least pressure value is noted at the tip of stenosis and balloon as compared to the inlet and outlet sections. In other graphs (4b-4j), the pressure value is minimum in the section containing the stenosis plus balloon as compared to the whole arterial segment. The pressure value is lower in this section since the flow is passing at a high speed through this section that is evident from velocity magnitude flow profile graphs. As the iterations move from t=1 to t=10, the pressure is decreasing in the outlet region that is the region which lies after the flow passes through the stenosis plus balloon section. The declining pressure in the succeeding plaque plus balloon locality shows that flow is slightly increasing as the fluid transits through the narrow zone developed due to stenosis and balloon. This increase in the velocity is also evident from velocity simulation outcomes that the flow is escalating as the flow passes through stenosis plus balloon region for t=1 to t=10. In these pressure graphs a higher-pressure outcome is disclosed at the inlet (Left Hand Side) in comparison to the outlet (Right Hand Side) of the coronary artery. Figures (5a-5e) present the streamline graphs for this endoscopy analysis. These streamline graphs are computed for t=1,3,5,7,8 respectively. In these streamline graphs, the utmost flow is evident at the crown of plaque assembly and balloon. Thus, a high velocity magnitude is observed due to the narrowing of channel caused due to stenosis plus balloon placement. It is observed that the flow is laminar, and no streamlines cross each other before the flow transits the stenosis plus balloon zone but as the flow transmits the narrow spot formed by stenosis plus balloon then it increases the flow velocity to an extent that one can already see a turbulence plus swirling effect as the flow leaves this stenosis plus balloon region. Thus, as the flow leaves the stenosis plus balloon region, there streamlines cross each other and a turbulence plus swirling effect is observed to some extent before the flow leaves this arterial segment again as a laminar flow. This turbulence occurs naturally in the realistic hemodynamics analysis for the plaque assembly problems that is also verified through our results.

## 5 Conclusions

The endoscopy of a coronary arterial segment having a symmetric plaque assembly in the innermost zone is numerically interpreted. Numerical simulations are covered for blood flow profile and pressure outcomes are also included. The major outcomes of this endoscopy analysis include the following points.

• The endoscopy of such stenosed sections is necessary before the process of angioplasty or bypass surgery since the endoscopy analysis provides the necessary data related to stenosis locality, size, height, formation etc that is required for the purpose of stent placement.
• The velocity profile simulations show that the flow entering the narrow section, that is formed due to the stenosis and balloon placement, attains a high velocity and the same flow profile declines for the flow leaving the narrow section but with increasing value of t to 10, a minor increase in the flow is examined after leaving the stenosis plus balloon section.
• The pressure profile shows a high value of pressure near the inlet region while a lower pressure value is illustrated about the outlet zone of arterial segment. The increasing values of t to 10 also results in a declined pressure profile near the outlet section.
• The stenosis and balloon over endoscope have made the channel so narrow that the flow leaving the stenosis plus balloon region faces some swirling and turbulent flow effects for some time and eventually the flow again becomes laminar before leaving the arterial segment.

## Data availability statement

The data used in the present manuscript is fully included within the manuscript.

## CRediT authorship contribution statement

**Salman Akhtar:** Conceptualization, Data curation, Formal analysis, Investigation, Methodology, Project administration, Resources, Software, Writing – original draft. **Zahir Hussain:** Conceptualization, Formal analysis, Project administration, Resources, Supervision, Validation, Visualization. **Hassan Ali Ghazwani:** Data curation, Formal analysis, Funding acquisition, Validation, Visualization, Writing – review & editing. **Showkat Ahmad Lone:** Data curation, Funding acquisition, Methodology, Resources, Validation, Writing – review & editing. **Emad A. Az-Zo'bi:** Data curation, Funding acquisition, Investigation, Methodology, Resources, Validation, Writing – review & editing.

## Declaration of competing interest

The authors declare that there is no conflict of interest.
